# Outpatient sexually transmitted infection testing and treatment patterns in the United States: a real-world database study

**DOI:** 10.1186/s12879-023-08434-2

**Published:** 2023-07-13

**Authors:** Rebecca Lillis, Louis Kuritzky, Zune Huynh, Rodney Arcenas, Avneet Hansra, Roma Shah, Baiyu Yang, Stephanie N. Taylor

**Affiliations:** 1 Section of Infectious Diseases, Department of Medicine, Louisiana State University Health Sciences Center, New Orleans, LA 70119 USA; 2 Department of Community Health and Family Medicine, University of Florida, Gainesville, FL USA; 3 Clinical Faculty, University of Central Florida/Hospital Corporation of America Family Medicine Residency, Gainesville, FL USA; 4 Roche Molecular Systems, Inc, Pleasanton, CA USA

**Keywords:** *Chlamydia trachomatis*, *Neisseria gonorrhoeae*, Diagnostic testing, Antimicrobial treatment, Sexually transmitted infections

## Abstract

**Background:**

*Chlamydia trachomatis* (CT) and *Neisseria gonorrhoeae* (NG) are the most common notifiable sexually transmitted infections (STIs) in the United States. Because symptoms of these infections often overlap with other urogenital infections, misdiagnosis and incorrect treatment can occur unless appropriate STI diagnostic testing is performed in clinical settings. The objective of this study was to describe STI diagnostic testing and antimicrobial treatment patterns and trends among adolescent and adult men and women with lower genitourinary tract symptoms (LGUTS).

**Methods:**

We analyzed insurance claims data from the IBM® MarketScan® Research Databases. Patients included were between 14 and 64 years old with LGUTS as determined by selected International Classification of Diseases codes between January 2010 and December 2019. Testing of STIs and relevant drug claims were captured, and distribution of testing patterns and drug claims were described.

**Results:**

In total, 23,537,812 episodes with LGUTS (87.4% from women; 12.6% from men) were analyzed from 12,341,154 patients. CT/NG testing occurred in only 17.6% of all episodes. For episodes where patients received treatment within 2 weeks of the visit date, 89.3% received treatment within the first 3 days (likely indicating presumptive treatment), and 77.7% received it on the first day. For women with pelvic inflammatory disease and men with orchitis/epididymitis and acute prostatitis, ≤ 15% received CT/NG testing, and around one-half received antibiotic treatment within 3 days.

**Conclusions:**

Our study revealed low CT/NG testing rates, even in patients diagnosed with complications commonly associated with these STIs, along with high levels of potentially inappropriate presumptive treatment. This highlights the need for timely and accurate STI diagnosis in patients with LGUTS to inform appropriate treatment recommendations.

**Supplementary Information:**

The online version contains supplementary material available at 10.1186/s12879-023-08434-2.

## Background

In 2018, there were an estimated 26.2 million new cases of sexually transmitted infections (STIs) in the United States (US), with almost one-half of these in 15–24 year-olds [1]. For the two most common notifiable STIs, *Chlamydia trachomatis* (CT) and *Neisseria gonorrhoeae* (NG), 1,644,416 CT cases and 710,151 NG cases were reported in the US in 2021 [2]. This was a 3.9% and 4.6% increase in CT and NG cases, respectively compared with the previous year [2] and is likely due to reduced STI screening during the COVID-19 pandemic resulting in undiagnosed CT infections in 2020 [3].

STIs have far-reaching public health consequences, therefore, effective diagnosis and appropriate treatment of STIs are required to support the prevention and control of these infections [4]. The US national strategic plan for STIs (2021–2025) emphasizes the importance of expanding the STI workforce and delivering STI services in all settings, especially primary care [5]. For the diagnosis of CT and NG, nucleic acid amplification tests (NAATs) are recommended, and culture can also be used for the diagnosis of NG [4, 6].

Complications due to NG in women include but are not limited to cervicitis [7], urethritis [7] and pelvic inflammatory disease (PID) [8], while infection in men can lead to urethritis [7] and epididymitis [9]. NG can also cause infection in extra-genital sites [7, 10]. Treatment of NG has historically involved presumptively administering antimicrobials before laboratory results are available and according to evidence-based management guidelines [11]. However, NG has developed antimicrobial resistance (AMR) to all drugs previously recommended for treatment of gonorrhoea [7]. Since 2020 in the US, a single dose of ceftriaxone is recommended for uncomplicated NG infection [4, 12]; however, strains of bacteria with high-level ceftriaxone resistance were identified in 2009, and since then, other resistant strains have emerged [7]. Appropriate antimicrobial stewardship efforts are therefore needed to ensure NG remains treatable.

For those diagnosed with CT, treatment should be provided promptly to reduce transmission and complications [4], such as cervicitis [13] or PID [8] in women, and epididymitis [9], epididymo-orchitis, urethritis or prostatitis in men [14]. Like NG, CT can also cause infections in extra-genital sites [10]. The current recommended treatment for CT is a 7-day course of doxycycline [4]. Despite the wide availability of effective drugs against CT and a lack of CT antibiotic resistance mechanisms, this pathogen continues to cause widespread persistent infections [15].

The symptoms of both CT and NG infections often overlap with other urogenital tract infections, therefore without suitable diagnostic tools these infections can be misdiagnosed and/or treated inappropriately [16]. Previous studies suggest that screening rates for STIs may not be optimal, with opportunities for improvements in primary care settings [17, 18]. It is therefore important to understand current patterns of STI testing and treatment to identify the greatest unmet needs.

The IBM® MarketScan® Research Databases provide one of the largest collections of proprietary de-identified claims data for privately and publicly insured people in the US [19]. The objective of this study was to analyze data from this database relating to patients that presented with lower genitourinary tract symptoms (LGUTS), which could be indicative of an STI. From these data, diagnostic testing and antimicrobial treatment patterns and trends were described to identify patients with the highest unmet diagnostic and treatment needs, and ultimately contribute to the improvement of patient management and outcomes.

## Methods

### Study design and population

This was a retrospective, observational study using anonymized data from the IBM MarketScan Research Databases (Commercial Database and Multi-State Medicaid Database). Patients included were between 14 and 64 years old and presented with International Classification of Diseases (ICD) codes indicative of signs, symptoms or diagnosis of a urogenital condition that could be caused by an STI between January 2010 and December 2019. The age range selected was chosen to capture those at highest risk of STIs. Previous research shows that incidence of STIs is particularly high in adolescents and young adults [20, 21], but has been increasing across all age groups, up to 64 years of age, in the US [22]. Patients who received prophylactic treatment as a contact to an infected partner and who were asymptomatic were not included.

### ICD codes and inclusion and exclusion criteria for the patient cohort

**Fig. 1:**
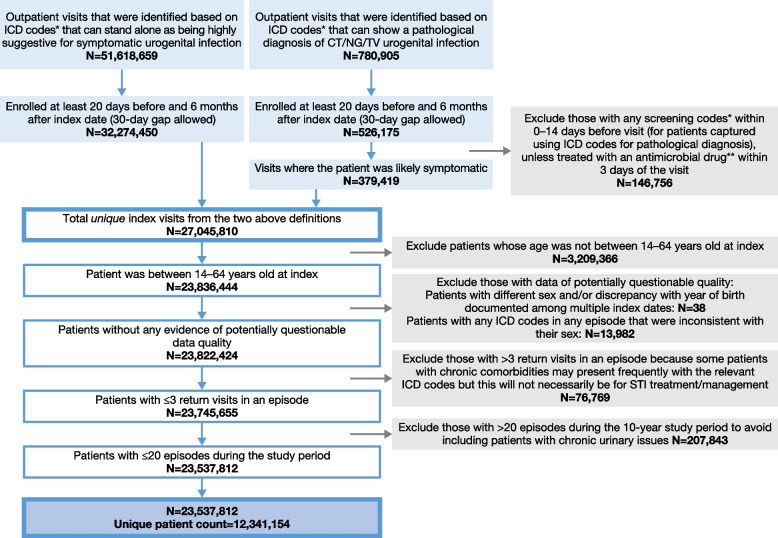
Flowchart of included patient episodes. *See Table S1; **See Table S3. *CT* *Chlamydia trachomatis*; *ICD* International Classification of Diseases, *N* Number, *NG* *Neisseria gonorrhoeae*, *TV* *Trichomonas vaginalis*

The inclusion and exclusion criteria for the cohort are shown in Fig. 1.

#### Inclusions

Patients were captured using ICD-9 or ICD-10 codes in outpatient claims (Additional file 1: Table S1), which could stand alone as being highly suggestive for symptomatic urogenital infection or show a clinical diagnosis of CT, NG, and/or *Trichomonas vaginalis* (TV) urogenital infection. The clinical diagnosis codes were added to avoid missing symptomatic patients due to variations in using ICD diagnosis codes to document symptoms. Additional exclusion criteria for this clinical diagnosis group were applied to only include patients that were likely symptomatic (see Exclusions section below).

#### Index date, episode and visit definitions

**Fig. 2:**
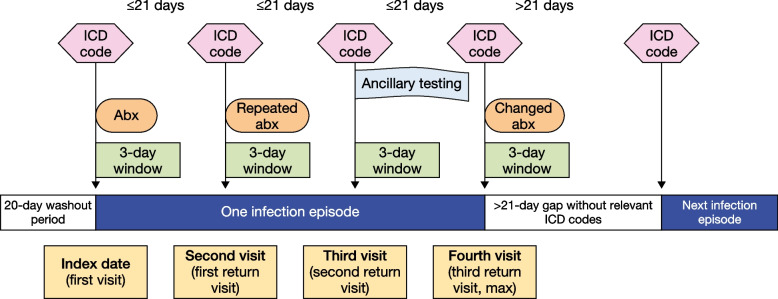
Diagram to demonstrate the definitions of symptomatic episode period and visit dates. *ICD* International Classification of Diseases; *abx* Antibiotic treatment

The index date is defined as the first date that any ICD code of interest was captured. If another ICD code of interest was captured within 21 days (inclusive of the index date), the infection was considered ongoing. However, if there was a gap of > 21 days without any code of interest, it was considered that the current infection had ended before the gap. As such, the episode is defined as the period between the index date and the last date before a gap of > 21 days without any ICD codes of interest (Fig. 2). This 21-day window was chosen to capture potential treatment failures and misdiagnoses from the index visit, which usually present within that timeframe.

The first visit was defined as the index date plus 2 more days to capture events related to the first visit (Fig. 2). This timeframe was selected as testing ordered on the index date may take up to 3 days to be billed, due to the time taken to log the specimen in the system. Treatment prescribed on the index date may also take up to 3 days before being ready for collection. Documentation of another ICD code of interest within 4–21 days of the index date indicates a return visit.

#### Exclusions

Patients from the inpatient setting were not considered, as their treatment pathway is different from the outpatient setting. Patients with < 20 days of continuous follow-up pre index date and/or < 6 months’ follow-up after the index date of each episode were excluded. The pre-index period was selected to make sure this index date was the beginning of a new episode and not a return visit. The post-index period was selected to allow enough time to capture the full course of an episode. For patients captured using ICD codes for clinical diagnosis, those with screening codes within 0–14 days before visit were excluded. This was to ensure only symptomatic patients were included and not patients who were diagnosed through screening and likely asymptomatic. However, if the patient received antibiotic treatment within 3 days of the visit, they were included to avoid missing patients who came in for screening or had a screening code but presented with symptoms and were managed acutely. Patients from the MarketScan Medicare Supplemental Database were excluded as they were aged ≥ 65 years old and not relevant to our study aims. Patients with probable inconsistent or inaccurate data (for example, diagnoses not consistent with sex such as epididymitis in an individual identifying as female), with > 3 return visits within an episode, and with > 20 episodes during the 10-year study period were excluded (Fig. 1).

### Codes for testing and antimicrobial use

All Current Procedural Terminology (CPT) codes for testing of CT, NG, *Mycoplasma*, TV, *bacterial vaginosis* (BV), herpes simplex virus, urinalysis, bacterial culture and others were included (Additional file 1: Table S2). Tests within 3 days of each visit were associated with that visit. Tests on day ≥ 4 of each visit were excluded unless they were associated with the next visit.

Antimicrobial drugs relevant to CT, NG, *Mycoplasma genitalium* (MG), TV, BV, and UTIs were captured (Additional file 1: Table S3). For antibiotic treatment, an extended 14-day window starting from each visit was selected to capture antibiotics prescribed after the initial visit but still associated with that visit, e.g., treatment prescribed after clinician receives test results.

### Data analysis

The distribution of STI testing practices of all episodes was stratified by age, sex, and year. The proportion of episodes with claims for each antibiotic class up to 14 days of the last visit date, stratified by year and drug class were described. One episode may have had more than one antimicrobial drug claim. Testing and antibiotic treatment (within 1–3 days) of patients with pertinent conditions for which guidelines recommend prompt diagnosis and treatment, stratified by age were described. The unit of analysis for this study was each infection episode.

Analyses were performed using SAS Studio, version 3.8.

## Results

### Episodes and patient characteristics

In total, 23,537,812 LGUTS episodes (87.4% from women; 12.6% from men) (Additional file 1: Table S4) were analyzed from 12,341,154 patients (Fig. 1). The median age of patients at index was 38 years old (interquartile range 26–51 years), with 46.2% of the cohort aged between 40 and 64 years old. Further patient demographics are shown in Additional file 1: Table S4.

### Diagnostic testing patterns and trends

Over the study period, only 17.6% of all episodes received CT/NG testing (Table 1). However, rates of CT/NG testing generally increased over time in all age groups (Additional file 1: Table S5). Those presenting with LGUTS who were most often tested for CT/NG were the 20–24-year-olds; testing occurred in 44.3% and 31.3% of episodes from men and women, respectively, in this group (Table 1). The 40–64-year age bracket was the least likely age group to receive CT/NG testing; only 7.8% and 7.4% of episodes in men and women aged ≥ 40, respectively, received testing. Those aged between 40 and 64 years old most often received non-CT/NG testing. Similar percentages of episodes that received no testing were observed across all age groups for men (18.6–22.9%) and women (12.2–15.8%).

**Table 1:** STI testing practices for all episodes stratified by age at index and sex

**Age group**	**All episodes**^a^ **N**	**Episodes with CT/NG testing**^b^ **N (%)**^c^	**Episodes with non-CT/NG testing**^**d**c^ **N (%)**	**Episodes with** **no testing** **N (%)**^c^
**Men**				
14–19	233,699	73,738 (31.6)	116,444 (49.8)	43,517 (18.6)
20–24	271,356	120,274 (44.3)	94,363 (34.8)	56,719 (20.9)
25–29	216,369	82,737 (38.2)	87,097 (40.3)	46,535 (21.5)
30–34	227,150	66,541 (29.3)	110,211 (48.5)	50,398 (22.2)
35–39	243,434	54,273 (22.3)	113,457 (54.8)	55,704 (22.9)
40–64	1,781,675	139,614 (7.8)	1,250,444 (70.2)	391,617 (22.0)
**Women**				
14–19	1,942,852	490,762 (25.3)	1,215,844 (62.6)	236,246 (12.2)
20–24	2,779,432	870,400 (31.3)	1,531,524 (55.1)	377,508 (13.6)
25–29	2,385,694	671,539 (28.1)	1,372,264 (57.5)	341,891 (14.3)
30–34	2,279,176	519,450 (22.8)	1,420,423 (62.3)	339,303 (14.9)
35–39	2,088,773	382,113 (18.3)	1,379,599 (66.0)	327,061 (15.7)
40–64	9,088,202	674,178 (7.4)	6,977,734 (76.8)	1,436,290 (15.8)
**All patients**				
All	23,537,812	4,145,619 (17.6)	15,689,404 (66.7)	3,702,789 (15.7)

*CT* Chlamydia trachomatis, *NG* Neisseria gonorrhoeae

^a^Testing within 1–3 days of index date and/or any return visits within each episode (date of visit as day 1)

^b^Includes episodes that received CT/NG testing only and episodes that received CT/NG testing and non-CT/NG testing

^c^All percentages are row percentages

^d^Episodes tested for urogenital infections other than CT and NG

### Evolution of antimicrobial therapy over time

Of all episodes included, 44.4% did not receive antibiotics between the index date and return visit or end of episodes. For those episodes in which patients ultimately received treatment within 2 weeks of the index date, 89.3% received treatment within the first 3 days, and 77.7% received it on the index date.

The largest percentage of antimicrobial claims over the study period was for urinary anti-infectives (Table 2a). Of all episodes, 24.7% had a urinary anti-infective claim within the entire episode including up to 14 days of the last visit date. When stratified by year, urinary anti-infective claims increased substantially from 20.8% in 2010 to 30.2% in 2019.

**Table 2:** Episodes with antimicrobial drug claims. A) all episodes^a^, b) all episodes among those with *Neisseria gonorrhoeae*^a^

	**Total episodes**	**Percentage**^**b**^** of episodes with antimicrobial drug claims**												
	**N**	** ≥ 1 drug claims**	**PEN**	**MAC**	**CEP**^c^	**CFM**	**CRO**	**FLQ**	**TET**	**UAI**	**NIM**	**GEN**	**ETP**	**CLI**
**a)**														
** All**	23,537,812	58.1	3.1	3.3	4.3	0.1	4.3	19.0	2.6	24.7	12.3	0.26	0.07	1.7
** 2010**	2,383,335	52.6	2.7	2.7	2.6	0.2	2.2	22.3	2.4	20.8	9.3	0.27	0.04	1.6
** 2011**	2,533,080	53.8	2.7	2.9	2.8	0.2	2.5	22.3	2.5	21.5	9.6	0.26	0.04	1.7
** 2012**	2,739,687	55.8	2.8	3.2	3.2	0.2	3.3	20.9	2.8	22.4	11.3	0.25	0.06	1.7
** 2013**	2,609,341	56.4	2.9	3.1	3.4	0.2	3.7	20.6	2.6	22.8	11.7	0.26	0.06	1.7
** 2014**	2,791,885	58.2	3.0	3.3	3.9	0.1	4.1	20.4	2.6	23.6	12.7	0.25	0.07	1.7
** 2015**	2,533,735	60.1	3.1	3.5	4.4	0.1	4.8	20.4	2.6	24.6	13.3	0.27	0.07	1.7
** 2016**	2,413,673	61.2	3.2	3.6	5.0	0.1	5.6	18.1	2.7	26.6	13.9	0.28	0.09	1.8
** 2017**	2,236,204	62.1	3.4	3.7	6.0	0.1	5.9	14.8	2.8	29.1	14.3	0.27	0.09	1.8
** 2018**	2,185,425	62.4	3.5	3.7	6.9	0.1	6.0	13.3	2.8	30.1	14.4	0.26	0.10	1.8
** 2019**	1,111,447	61.7	3.6	3.8	7.7	0.1	6.5	11.1	2.8	30.2	14.3	0.24	0.12	1.7
**b)**														
** All**	60,005	66.4	1.5	24.3	1.3	1.1	46.0	3.4	8.1	3.2	11.1	0.18	0.01	2.0
** 2010**	3,594	58.3	1.4	17.1	1.2	2.6	31.5	7.1	13.3	3.5	10.2	0.11	0.00	1.1
** 2011**	4,003	62.4	1.5	19.6	1.6	2.3	37.0	6.2	13.2	3.1	9.4	0.02	0.00	1.2
** 2012**	7,298	65.8	1.4	24.1	1.3	1.9	44.8	3.9	8.8	3.0	10.1	0.11	0.00	0.9
** 2013**	7,337	66.3	1.4	24.9	1.1	1.1	46.1	3.3	9.3	3.1	10.4	0.10	0.00	1.0
** 2014**	9,995	66.2	1.5	25.0	1.0	0.7	46.1	2.8	7.8	2.9	10.2	0.06	0.03	3.2
** 2015**	9,262	64.4	1.7	24.2	1.3	0.6	44.7	3.1	5.8	3.0	10.6	0.08	0.00	3.5
** 2016**	5,809	66.9	1.5	23.2	1.6	0.8	48.4	2.4	6.5	3.2	12.5	0.28	0.03	1.4
** 2017**	5,792	69.3	1.6	25.7	1.6	0.7	49.2	2.4	6.3	3.8	14.7	0.38	0.02	2.7
** 2018**	4,505	73.4	1.6	30.1	1.4	0.7	55.1	2.1	6.7	3.7	12.5	0.40	0.00	1.2
** 2019**	2,410	73.6	1.7	28.5	1.8	0.9	59.5	2.6	6.2	2.8	12.4	0.91	0.00	1.0

*CFM* Cefixime, *CRO* Ceftriaxone, *CEP* Cephalosporin, *CLI* clindamycin, *ETP* ertapenem, *FLQ* fluoroquinolone, *GEN* Gentamicin, *MAC* Macrolide, *NIM* Nitroimidazole, *PEN* Penicillin, *TET* Tetracycline, *UAI* Urinary anti-infectives

^a^Stratified by year and drug class within the entire episode including up to 14 days of the last visit date

^b^All percentages are row percentages

^c^CEP group excludes CRO and CFM as these cephalosporins were considered separately due to their recommended use as per NG treatment guidelines

Of antimicrobials prescribed, the second highest claims were for fluoroquinolones (ciprofloxacin, levofloxacin, moxifloxacin, ofloxacin) (19.0%) and there was a 18% relative reduction for fluoroquinolone claims between 2016 and 2017 from 18.1% to 14.8%, respectively (Table 2a), reflective of the Food and Drug Administration restriction in 2016 [23].

Of all episodes, 12.3% had nitroimidazole (e.g., metronidazole) claims within the entire episode including up to 14 days of the last visit date. Ceftriaxone claims gradually increased from 2010 to 2019 (2.2–6.5%; Table 2a), reflective of the changes to the Centers for Disease Control and Prevention (CDC) STI treatment guidelines [12].

Only 66.4% of all episodes in patients diagnosed with NG had an associated antibiotic claim within the entire episode including up to 14 days of the last visit date (Table 2b). From 2010 to 2018, there was a 73% relative reduction for cefixime claims (2.6% to 0.7%) and between 2010 and 2012 these claims fell by 26% (2.6% to 1.9%), indicative of decreased susceptibility of NG strains [24] to cefixime in the US and changes to the STI treatment guidelines in that timeframe [25]. Macrolide, ceftriaxone, and gentamicin claims for those diagnosed with NG generally increased from 2010 to 2019.

### Testing and treatment patterns based on specific diagnostic codes

For women diagnosed with PID, 15.0% were tested for CT/NG and 41.9% were prescribed antibiotics within 3 days of diagnosis (Table 3). The age group most and least likely to receive CT/NG testing (days 1–3) were 14–19-year-olds (30.2%) and 40–64-year-olds (7.9%), respectively. The age group most and least likely to receive treatment (days 1–3) were 20–24-year-olds (57.1%) and 40–64-year-olds (28.4%), respectively. Of those women diagnosed with cervicitis, 24.2% received CT/NG testing and 17.1% received antibiotic treatment on days 1–3. The age group most and least likely to receive CT/NG testing and treatment within 3 days were 14–19-year-olds (64.8% tested; 41.9% treated) and 40–64-year-olds (10.3% tested; 10.9% treated), respectively.

**Table 3:** Testing and treatment patterns of patients with certain diagnoses on day 1, stratified by age

Age group	All episodes	Received CT/NG test (days 1–3) N (%)^a^	Received antibiotic treatment (days 1–3) N (%)	Received CT/NG test and/or antibiotic treatment (days 1–3) N (%)
**Women with PID on day 1 (index)**				
All	13,232	1,990 (15.0)	5,549 (41.9)	6,099 (46.1)
14–19	486	147 (30.2)	277 (57.0)	322 (66.3)
20–24	1,565	426(27.2)	893 (57.1)	995 (63.6)
25–29	1,767	381 (21.6)	994 (56.3)	1,086 (61.5)
30–34	2,121	380 (17.9)	1,058 (49.9)	1,155 (54.5)
35–39	2,009	239 (11.9)	828 (41.2)	891 (44.4)
40 +	5,284	417 (7.9)	1,499 (28.4)	1,650 (31.2)
**Women with cervicitis on day 1 (index)**				
All ages	839,029	203,183 (24.2)	143,175 (17.1)	269,806 (32.2)
14–19	35,578	23,037 (64.8)	14,920 (41.9)	26,825 (75.4)
20–24	110,417	51,504 (46.6)	28,981 (26.2)	59,949 (54.3)
25–29	116,082	39,845 (34.3)	23,657 (20.4)	48,344 (41.6)
30–34	115,109	30,413 (26.4)	20,139 (17.5)	39,465 (34.3)
35–39	109,108	22,025 (20.2)	17,148 (15.7)	31,442 (28.8)
40 +	352,735	36,359 (10.3)	38,330 (10.9)	63,781 (18.1)
**Men with orchitis/epididymitis on day 1 (index)**				
All	390,440	40,149 (10.3)	227,290 (58.2)	236,073 (60.5)
14–19	31,396	5,400 (17.2)	18,373 (58.5)	19,628 (62.5)
20–24	38,583	8,362 (21.7)	23,149 (60.0)	25,154 (65.2)
25–29	32,610	5,738 (17.6)	19,385 (59.4)	20,730 (63.6)
30–34	40,106	5,363 (13.4)	24,079 (60.0)	25,292 (63.1)
35–39	45,404	4,425 (9.7)	27,233 (60.0)	28,146 (62.0)
40 +	202,341	10,861 (5.4)	115,071 (56.9)	117,123 (57.9)
**Men with urethritis on day 1 (index)**				
All	250,288	144,384 (57.7)	154,031 (61.5)	202,042 (80.7)
14–19	29,061	17,691 (60.9)	17,460 (60.1)	23,317 (80.2)
20–24	54,758	35,347 (64.6)	33,980 (62.1)	45,877 (83.8)
25–29	38,857	24,518 (63.1)	24,061 (61.9)	32,419 (83.4)
30–34	30,657	18,651 (60.8)	19,425 (63.4)	25,494 (83.2)
35–39	24,727	14,402 (58.2)	15,537 (62.8)	20,229 (81.8)
40 +	72,228	33,775 (46.8)	43,568 (60.3)	54,706 (75.7)
**Men with acute prostatitis on day 1 (index)**				
All	215,315	8,531 (4.0)	123,762 (57.5)	125,621 (58.3)
14–19	1,356	175 (12.9)	856 (63.1)	896 (66.1)
20–24	5,580	795 (14.2)	3,361 (60.2)	3,535 (63.4)
25–29	7,254	940 (13.0)	4,317 (59.5)	4,533 (62.5)
30–34	11,600	1,144 (9.9)	7,123 (61.4)	7,377 (63.6)
35–39	16,190	1,196 (7.4)	9,854 (60.9)	10,137(62.6)
40 +	173,335	4,281 (2.5)	98,251 (56.7)	99,143 (57.2)

*CT* Chlamydia trachomatis, *NG* Neisseria gonorrhoeae, *PID* pelvic inflammatory disease

^a^All percentages are row percentages

For men diagnosed with orchitis/epididymitis, urethritis, and acute prostatitis, 10.3%, 57.7%, and 4.0% received CT/NG testing, respectively, and 58.2%, 61.5%, and 57.5% received antibiotic treatment within 3 days of diagnosis, respectively (Table 3). For all three male-specific diagnoses, the age groups most and least likely to receive CT/NG testing within 3 days were the 20–24-year-olds and 40–64-year-olds respectively, and treatment rates were similar across all age groups.

Testing rates for these specific conditions were similar when extending the time period up to 7 days after diagnosis, therefore almost all testing occurred within 1–3 days (data not shown).

## Discussion

This study has revealed that from 2010–2019, despite patients presenting with signs and symptoms consistent with and/or suggestive of STIs, < 20% of all these episodes had CT/NG testing, which suggests diagnostic testing for CT/NG is being underutilized. CT/NG testing did generally increase over time in all age groups for men and women, which could potentially be due to increasing availability of NAAT tests that can be performed using less invasive self-collected urine or vaginal specimens [6, 26]. Over the study period, 20–24 year-olds had the highest rates of testing, which is not surprising considering that this age group has the highest rates of NG infection in men and women and CT infection in women [6]. However, testing rates in the ≤ 24 year-olds were still low considering young people are at greater risk of STIs [27]. Those 40–64 years old had the lowest testing rates, possibly due to lower rates of CT/NG infections in this group [28] and the consideration of other pathologies potentially causing LGUTS. However, rates of these STIs are increasing in people over the age of 40 [28] and thus discussion of sexual history and STI testing should continue to be considered when assessing people of any age group presenting with LGUTS.

Of all episodes that received antibiotic treatment within 2 weeks, the majority (89.3%) received treatment within the first 3 days, which is assumed to be presumptive therapy, due to the time it could take to receive testing and obtain results. The extensive prescription of antibiotic treatment within 3 days is often unavoidable when test results are unavailable, and their use suggests clinicians are prescribing treatments based on their presumptive diagnosis, rather than waiting for test results. This presumptive treatment may contribute to suboptimal antimicrobial stewardship. A disparity in testing and treatment rates was noted for women diagnosed with PID and men diagnosed with orchitis/epididymitis and acute prostatitis, with ≤ 15% receiving CT/NG testing, and around one-half of patients receiving antibiotic treatment within 3 days. These data are concerning, given that both CT and NG can cause these diseases and associated complications [7–9, 13, 14]. Even though the higher treatment rates compared with testing rates could indicate inappropriate treatment, these treatment rates are still low considering empirical treatment regimens are recommended for certain groups with these conditions [4].

Our data suggest that the CDC testing guidelines, which state that all women with acute PID and cervicitis and men with urethritis and acute epididymitis should be tested for NG and CT [4, 29], were not being followed. Failure to test for CT/NG in these clinical syndromes would also imply a lack of partner notification, as these patients would be unable to inform their partner of their diagnosis. Untreated partners can lead to reinfection and potential complications for the index patient.

Claims associated with antimicrobials fluctuated over the study period, likely due to changes in treatment guidelines. Decreasing use of fluoroquinolone between 2016 and 2017 is attributed to the 2016 updated U.S. Food and Drug Administration guidelines that recommended restricting fluoroquinolone use in patients with uncomplicated UTIs [23]. The largest percentage of antimicrobial claims was for urinary anti-infectives, which reflects the treatment of UTIs as these antibiotics are not typically used to treat STIs. However, dysuria is a common STI symptom [6] and if this patient group were treated presumptively and not tested for STIs, there may have been missed STI diagnoses. The decline in cefixime claims for those diagnosed with NG are reflective of the 2012 changes to the 2010 CDC treatment guidelines, which no longer recommended oral cephalosporins, including cefixime, for NG treatment [25]. This has resulted in an increased reliance on ceftriaxone treatment. The guideline change was announced due to the rise in NG isolates with elevated minimum inhibitory concentrations to cefixime [25]. The large proportion of nitroimidazole claims likely reflected diagnoses of TV, BV, or both, since metronidazole or one of its derivatives is prescribed for these indications in patients with LGUTS [4].

Our study had some limitations. Assumptions were made to determine the exclusion and inclusion criteria and capture key events (e.g., return visit) for this study; however, the assumptions were carefully based on the consensus of practicing clinicians’ best judgement. Nonspecific STI symptoms could not be included e.g., abdominal pain, as it was not feasible to determine whether these symptoms were related to STIs. A large proportion of race and ethnicity data were missing or unreported and information about patients’ sexual practices and gender of partners was unavailable, therefore testing and treatment trends across different patient populations, such as men who have sex with men and gender diverse individuals, could not be observed. Throughout our study we use the terminology men/women based on the sex documented in the database, however it must be noted that those whose sex did not align with the diagnostic code were excluded, which subsequently excluded gender diverse individuals. Another limitation was that the number of enrollees in the IBM MarketScan Research Databases decreased after 2015 due to loss of data contributors, especially in 2018 and 2019. Our study also excluded patients diagnosed in the second half of 2019, because data were only available until the end of 2019 at the time of this analysis, and a 6-month follow-up period after index was required. Therefore, trends in 2018 and 2019 should be interpreted with caution. Finally, the validity of utilizing ICD codes for diagnosis of CT and NG has not been confirmed [30], thus the reliability of using claims data, including MarketScan, for surveillance research is unknown. It is possible that reported diagnostic codes may not capture all complaints and diagnoses [31]. However, findings based on diagnostic codes from claims data could indicate trends occurring across several healthcare systems within the database.

A previous call-to-action study iterated the importance of integrating accurate, rapid, affordable and accessible point-of-care (POC) STI tests into health systems to mitigate transmission and the burden of STIs [32]. Antimicrobial stewardship interventions are also needed for NG due to increasing AMR [33]. Improvements to diagnostic testing, and the emergence of POC tests, along with those that can determine antibiotic susceptibility, are crucial to support immediate diagnosis and appropriate STI treatment [26, 34]. Tests that enable self-collected samples could potentially improve testing rates, with one study showing that 70% of women receiving CT screening preferred to collect vaginal self-swabs if a POC was available [35]. Reporting the number of infections identified through STI testing is important to support public health authorities with allocation of resources, partner notification and treatment [36], although these rates will not be accurate if there is a lack of testing.

## Conclusion

Our study analyzing over 23 million LGUTS episodes suggestive of STIs revealed low levels of NG and CT testing, even in patients diagnosed with symptoms or conditions commonly associated with these STIs, along with high levels of antimicrobial treatment within 3 days of presenting with LGUTS, irrespective of whether CT/NG testing had occurred. Overall, this study highlights the need for rapid and accurate STI diagnosis in patients presenting with LGUTS to inform appropriate treatment recommendations.

## Supplementary Information

**Additional file 1: Table S1.** ICD codes for cohort definition. **Table S2.** CPT codes for testing related to signs and symptoms of urogenital infections. **Table S3.** Antimicrobial drugs. **Table S4.** Patient demographics. **Table S5.** STI testing patterns of all episodes over the different index years, stratified by age at index, among a) men and b) women

## Data Availability

The patient data that support the findings of this study are available from the IBM MarketScan Research Databases (Commercial Database and Multi-State Medicaid Database), but restrictions apply to the availability of these data, which were used under license for the current study and are not publicly available. The databases are available for secondary use on a commercial basis and requests for access to the data should be sent to IBM Watson Health and not the corresponding author. All data generated from analysis of the databases are included in this published article and its supplementary files.

## Abbreviations

AMR: Antimicrobial resistance
BV: *Bacterial vaginosis*
CDC: Centers for Disease Control and Prevention
CPT: Current Procedural Terminology
CT: *Chlamydia trachomatis*
ICD: International Classification of Diseases
LGUTS: Lower genitourinary tract symptoms
MG: *Mycoplasma genitalium*
NAATs: Nucleic acid amplification tests
NG: *Neisseria gonorrhoeae*
PID: Pelvic inflammatory disease
POC: Point-of-care
STIs: Sexually transmitted infections
TV: *Trichomonas vaginalis*
US: United States
