# Supplementary material for: Outpatient sexually transmitted infection testing and treatment patterns in the United States: a real-world database study
Source: BMC Infect Dis. 2023 Jul 13;23:469. doi: 10.1186/s12879-023-08434-2 (PMC10339584; doi:10.1186/s12879-023-08434-2)
Supplement: Supplementary file 1 — Additional file 1: Table S1. ICD codes for cohort definition. Table S2. CPT codes for testing related to signs and symptoms of urogenital infections. Table S3. Antimicrobial drugs. Table S4. Patient demographics. Table S5. STI testing patterns of all episodes over the different index years, stratified by age at index, among a) men and b) women [file 12879_2023_8434_MOESM1_ESM.docx]

**Additional file 1. Supplementary information**

**Manuscript title:**  Outpatient sexually transmitted infection testing and treatment patterns in the United States: a real-world database study

**Authors:** Rebecca Lillis, Louis Kuritzky, Zune Huynh, Rodney Arcenas, Avneet Hansra, Roma Shah, Baiyu Yang, Stephanie N. Taylor

# Table S1 ICD codes for cohort definition

| **Codes that stand alone as being highly suggestive for urogenital infection** | | | | |
| --- | --- | --- | --- | --- |
| **Category** | **ICD 10 codes** | **Description** | **ICD 9 codes** | **Description** |
| Acute cystitis | N30.0x* | Acute cystitis (with or without hematuria) | 595.0 | Acute cystitis |
| Urethritis | N34.1 | Nonspecific urethritis | 597.80 | Urethritis, unspecified |
|  |  |  | 099.40 | Other nongonococcal urethritis, unspecified |
|  |  |  | 099.41 | Other nongonococcal urethritis, chlamydia trachomatis |
|  |  |  | 099.49 | Other nongonococcal urethritis, other specified organism |
|  | N34.2 | Other urethritis | 597.89 | Other urethritis |
| Urethral syndrome | N34.3 | Urethral syndrome, unspecified | 597.81 | Urethral syndrome NOS |
| UTI, site not specified | N39.0 | Urinary tract infection, site not specified | 599.0 | Urinary tract infection, site not specified |
| Acute prostatitis | N41.0 | Acute prostatitis | 601.0 | Acute prostatitis |
| Prostatocystitis | N41.3 | Prostatocystitis | 601.3 | Prostatocystitis |
| Orchitis and epididymitis | N45.1 | Epididymitis | 604.90  604.99 | Orchitis and epididymitis, unspecified  Other orchitis, epididymitis, and epididymo-orchitis, without mention of abscess |
|  | N45.2 | Orchitis |  |  |
|  | N45.3 | Epididymo-orchitis |  |  |
| Acute salpingitis and oophoritis | N70.0x | Acute salpingitis and oophoritis | 614.0 | Acute salpingitis and oophoritis |
| Acute inflammatory disease of uterus [PID] | N71.0 | Acute inflammatory disease of uterus | 615.0 | Acute inflammatory diseases of uterus, except cervix |
| Inflammatory disease of cervix uteri [cervicitis] | N72 | Inflammatory disease of cervix uteri | 616.0 | Cervicitis and endocervicitis |
| Other female pelvic inflammatory diseases | N73.8 | Other specified female pelvic inflammatory diseases | 614.8 | Other specified inflammatory disease of female pelvic organs and tissues |
|  | N73.9 | Female pelvic inflammatory disease, unspecified | 614.9 | Unspecified inflammatory disease of female pelvic organs and tissues |
| Vaginitis, vulvitis, and vulvovaginitis | N76.0 | Acute vaginitis | 616.10 | Vaginitis and vulvovaginitis, unspecified |
|  | N76.2 | Acute vulvitis |  |  |
|  | N77.1 | Vaginitis, vulvitis and vulvovaginitis in infectious and parasitic diseases classified elsewhere | 616.11 | Vaginitis and vulvovaginitis in diseases classified elsewhere |
| Dysuria | R30.0 | Dysuria | 788.1 | Dysuria |
|  | R30.9 | Painful micturition, unspecified |  |  |
| Urethral discharge | R36.0 | Urethral discharge without blood | 788.7 | Urethral discharge |
|  | R36.9 | Urethral discharge, unspecified |  |  |
| **Codes showing a pathological diagnosis of CT, NG or TV infection in the urogenital system** | | | | |
| **Category** | **ICD 10 codes** | **Description** | **ICD 9 codes** | **Description** |
| CT infection | A56.0x | Chlamydial infection of lower genitourinary tract | 099.53 | Other venereal diseases due to *Chlamydia trachomatis*, lower genitourinary sites |
|  | A56.1x | Chlamydial infection of pelviperitoneum and other genitourinary organs | 099.54 | Other venereal diseases due to *Chlamydia trachomatis*, other genitourinary sites |
|  | A56.2 | Chlamydial infection of genitourinary tract, unspecified | 099.55 | Other venereal diseases due to *Chlamydia trachomatis*, unspecified genitourinary site |
| NG infection | A54.0x | Gonococcal infection of lower genitourinary tract without periurethral or accessory gland abscess | 098.0 | Gonococcal infection (acute) of lower genitourinary tract |
|  | A54.1 | Gonococcal infection of lower genitourinary tract with periurethral and accessory gland abscess |  |  |
|  | A54.2x | Gonococcal pelviperitonitis and other gonococcal genitourinary infection | 098.1x | Gonococcal infection (acute) of upper genitourinary tract |
| TV infection | A59.0x | Urogenital trichomoniasis | 131.0x | Urogenital trichomoniasis |

*x stands for all sub-codes

CT, *Chlamydia trachomatis*; ICD, International Classification of Diseases; NG, *Neisseria gonorrhoeae;* TV, *Trichomonas vaginalis*

# Table S2 CPT codes for testing related to signs and symptoms of urogenital infections

| **Category** | **Codes** | **Description** |
| --- | --- | --- |
| CT | 86631 | Analysis for antibody to CT |
|  | 86632 | Analysis for antibody (IgM) to CT |
|  | 87110 | Bacterial culture for CT |
|  | 87270 | CT antigen detection using immunofluorescent technique |
|  | 87320 | CT antigen detection using qualitative or semiquantitative multiple-step immunoassay technique |
|  | 87490 | CT detection by nucleic acid using direct probe technique |
|  | 87491 | CT detection by nucleic acid using amplified probe technique |
|  | 87492 | CT quantification by nucleic acid |
|  | 87810 | CT antigen detection by immunoassay with direct optical observation |
|  | 87800 | Infectious agent detection by nucleic acid (DNA or RNA), multiple organisms; direct probe(s) technique |
|  | 87801 | Infectious agent detection by nucleic acid (DNA or RNA), multiple organisms; direct probe(s) technique |
| NG | 87590 | NG detection by nucleic acid using direct probe technique |
|  | 87591 | NG detection by nucleic acid using amplified probe technique |
|  | 87592 | NG quantification by nucleic acid |
|  | 87850 | NG antigen detection by immunoassay with direct optical observation |
|  | [87800](https://www.uhcprovider.com/content/dam/provider/docs/public/policies/medadv-guidelines/s/screening-stis-behavioral-counseling-to-prevent.pdf) | Infectious agent detection by nucleic acid (DNA or RNA), multiple organisms; direct probe(s) technique |
|  | [87801](http://stdtac.org/wp-content/uploads/2016/05/ACA-Preventive-Services_STDTAC.pdf) | Infectious agent detection by nucleic acid (DNA or RNA), multiple organisms; amplified probe(s) technique |
| *Mycoplasma*  (not MG specific) | 87109 | Bacterial culture for *Mycoplasma* |
|  | 86738 | Analysis for antibody to *Mycoplasma* (bacteria) |
|  | 87798 | Infectious agent detection by nucleic acid (DNA or RNA), not otherwise specified; amplified probe technique, each organism |
| TV | 87660 | TV detection by nucleic acid using direct probe technique |
|  | 87661 | TV detection by nucleic acid using amplified probe technique |
|  | 87808 | TV antigen detection by immunoassay with direct optical observation |
| BV | 87510 | Infectious agent detection by nucleic acid (DNA or RNA); *Gardnerella vaginalis*, direct probe technique |
|  | 87511 | Infectious agent detection by nucleic acid (DNA or RNA); *Gardnerella vaginalis*, amplified probe technique |
|  | 87512 | Infectious agent detection by nucleic acid (DNA or RNA); *Gardnerella vaginalis*, quantification |
| Herpes simplex virus | 87273 | Infectious agent antigen detection by immunofluorescent technique; Herpes simplex virus type 2 |
|  | 87274 | Infectious agent antigen detection by immunofluorescent technique; Herpes simplex virus type 1 |
|  | 87528 | Infectious agent detection by nucleic acid (DNA or RNA); Herpes simplex virus, direct probe technique |
|  | 87529 | Infectious agent detection by nucleic acid (DNA or RNA); Herpes simplex virus, amplified probe technique |
|  | 87530 | Infectious agent detection by nucleic acid (DNA or RNA); Herpes simplex virus, quantification |
| Urinalysis | 81000 | Manual urinalysis test with examination using microscope |
|  | 81001 | Automated urinalysis using tablet reagent and microscopy of urine |
|  | 81002 | Manual urinalysis using dip stick |
|  | 81003 | Automated urinalysis using dip stick |
|  | 81005 | Urinalysis; qualitative or semiquantitative, except immunoassays |
|  | 81007 | Urinalysis; bacteriuria screen, except by culture or dipstick |
|  | 81015 | Urinalysis using microscope |
|  | 81020 | Urinalysis, 2 or 3 glass test |
|  | 81099 | Urinalysis procedure (unlisted) |
| Culture | 87040 | Aerobic and anaerobic bacterial culture of blood with isolation and presumptive identification of isolate |
|  | 87045 | Culture, bacterial; stool, aerobic, with isolation and preliminary examination (e.g., KIA, LIA), *Salmonella* and *Shigella* species |
|  | 87046 | Culture, bacterial; stool, aerobic, additional pathogens, isolation and presumptive identification of isolates, each plate |
|  | 87070 | Culture, bacterial; any other source except urine, blood or stool, aerobic, with isolation and presumptive identification of isolates |
|  | 87071 | Culture, bacterial; quantitative, aerobic with isolation and presumptive identification of isolates, any source except urine, blood or stool |
|  | 87073 | Culture, bacterial; quantitative, anaerobic with isolation and presumptive identification of isolates, any source except urine, blood or stool |
|  | 87075 | Culture, bacterial; any source, except blood, anaerobic with isolation and presumptive identification of isolates |
|  | 87076 | Bacterial culture for anaerobic isolates |
|  | 87077 | Bacterial culture for aerobic isolates |
|  | 87081 | Culture, presumptive, pathogenic organisms, screening only |
|  | 87086 | Bacterial colony count, urine |
|  | 87088 | Bacterial culture of urine |
| Other | 87205 | Smear, primary source with interpretation; Gram or Giemsa stain for bacteria, fungi, or cell types |
|  | 87210 | Wet mount smear from primary source with interpretation |
|  | 87300 | Infectious agent antigen detection by immunofluorescent technique, polyvalent for multiple organisms, each polyvalent antiserum |
|  | 87449 | Infectious agent antigen detection by immunoassay technique, qualitative or semiquantitative; multiple-step method, not otherwise specified, each organism |
|  | 87450 | Infectious agent antigen detection by immunoassay technique, qualitative or semiquantitative; single step method, not otherwise specified, each organism |
|  | 87451 | Infectious agent antigen detection by immunoassay technique, qualitative or semiquantitative; multiple step method, polyvalent for multiple organisms, each polyvalent antiserum |
|  | 87797 | Infectious agent detection by nucleic acid (DNA or RNA), not otherwise specified; direct probe technique, each organism |
|  | 87799 | Infectious agent detection by nucleic acid (DNA or RNA), not otherwise specified; quantification, each organism |
|  | 88141 | Cytopathology, cervical or vaginal (any reporting system), requiring interpretation by physician |
|  | 88142 | Cytopathology, cervical or vaginal (any reporting system), collected in preservative fluid, automated thin layer preparation; manual screening under physician supervision |
|  | 88175 | Cytopathology, cervical or vaginal (any reporting system), collected in preservative fluid, automated thin layer preparation; with screening by automated system and manual rescreening or review, under physician supervision |

BV, *bacterial vaginosis*; CPT, Current Procedural Terminology; CT, *Chlamydia trachomatis*; MG, *Mycoplasma genitalium*; NG, *Neisseria gonorrhoeae;* TV, *Trichomonas vaginalis*

# Table S3 Antimicrobial drugs

| **Drug class** | **Drug name** | **Treated infection** | | | | | | **Injection*** |
| --- | --- | --- | --- | --- | --- | --- | --- | --- |
|  |  | **CT** | **NG** | **MG** | **TV** | **BV** | **UTI** |  |
| Gentamicin | Gentamicin |  | x |  |  |  | x | x |
| Cefixime | Cefixime |  | x |  |  |  | x |  |
| Ceftriaxone | Ceftriaxone |  | x |  |  |  | x | x |
| Cephalosporin‡ | Cefadroxil |  |  |  |  |  | x |  |
|  | Cefdinir |  |  |  |  |  | x |  |
|  | Cefotaxime |  | x |  |  |  |  | x |
|  | Cefotetan |  |  |  |  |  | x | x |
|  | Cefoxitin |  | x |  |  |  |  | x |
|  | Cefpodoxime |  |  |  |  |  | x |  |
|  | Cephalexin |  |  |  |  |  | x |  |
| Fluoroquinolone | Ciprofloxacin |  | x |  |  |  | x |  |
|  | Levofloxacin | x | x |  |  |  | x |  |
|  | Moxifloxacin |  |  | x |  |  |  |  |
|  | Ofloxacin | x | x |  |  |  |  |  |
| Lincosamide | Clindamycin† |  |  |  |  | x |  |  |
| Macrolide | Azithromycin | x | x | x |  |  |  |  |
|  | Clarithromycin |  |  | x |  |  |  |  |
|  | Erythromycin | x |  | x |  |  |  |  |
|  | Fidaxomicin |  |  | x |  |  |  |  |
| Nitroimidazole | Metronidazole† |  |  |  | x | x |  |  |
|  | Tinidazole |  |  |  | x | x |  |  |
| Other | Ertapenem |  | x |  |  |  | x | x |
| Penicillin | Amoxicillin | x |  |  |  |  | x |  |
|  | Amoxicillin-clavulanate |  |  |  |  |  | x |  |
| Tetracycline | Doxycycline | x | x | x |  |  |  |  |
|  | Minocycline |  |  | x |  |  |  |  |
| Urinary anti-infective | Fosfomycin |  |  |  |  |  | x |  |
|  | Nitrofurantoin |  |  |  |  |  | x |  |
|  | Trimethoprim / Sulfamethoxazole (TMP/SMX) |  |  |  |  |  | x |  |
|  | Trimethoprim |  |  |  |  |  | x |  |

*In addition to National Drug Codes for oral drugs, Healthcare Common Procedure Coding System codes for injection for cefotaxime, cefotetan, cefoxitin, ceftriaxone, ertapenem, and gentamicin were included. †Intravaginal gel or cream for clindamycin and metronidazole were included. ‡Cephalosporin group excludes cefixime and ceftriaxone as these cephalosporins were considered separately due to their recommended use as per NG treatment guidelines.

BV, *bacterial vaginosis*; CT, *Chlamydia trachomatis*; MG, *Mycoplasma genitalium*; NG, *Neisseria gonorrhoeae;* TV, *Trichomonas vaginalis*; UTI, urinary tract infections

# Table S4 Patient demographics

|  | **All**  **N (%)** | **Men**  **N (%)** | **Women**  **N (%)** |
| --- | --- | --- | --- |
| **Total episodes** | 23,537,812 (100.0) | 2,973,683 (100.0) | 20,564,129 (100.0) |
| **Sex** |  |  |  |
| Men | 2,973,683 (12.6) | 2,973,683 (100.0) | 0 (0.0) |
| Women | 20,564,129 (87.4) | 0 (0.0) | 20,564,129 (100.0) |
| **Age at index, years** |  |  |  |
| 14–19 | 2,176,551 (9.2) | 233,699 (7.9) | 1,942,852 (9.4) |
| 20–24 | 3,050,788 (13.0) | 271,356 (9.1) | 2,779,432 (13.5) |
| 25–29 | 2,602,063 (11.1) | 216,369 (7.3) | 2,385,694 (11.6) |
| 30–34 | 2,506,326 (10.6) | 227,150 (7.6) | 2,279,176 (11.1) |
| 35–39 | 2,332,207 (9.9) | 243,434 (8.2) | 2,088,773 (10.2) |
| 40–64 | 10,869,877 (46.2) | 1,781,675 (59.9) | 9,088202 (44.2) |
| **Age at index, years** |  |  |  |
| Mean (SD) | 38.5 (14.3) | 42.8 (14.8) | 37.8 (14.1) |
| Median (IQR) | 38.0 (26.0–51.0) | 45.0 (30.0–56.0) | 37.0 (25.0–50.0) |
| **Insurance** |  |  |  |
| Commercial | 18,701,619 (79.5) | 2,567,958 (86.4) | 16,133,661 (78.5) |
| Medicaid | 4,836,193 (20.5) | 405,725 (13.6) | 4,430,468 (21.5) |
| **Place of service** |  |  |  |
| Urgent care | 1,149,493 (4.9) | 133,748 (4.5) | 1,015,745 (4.9) |
| Emergency room visits | 2,665,005 (11.3) | 350,661 (11.8) | 2,314,344 (11.3) |
| Non-ER outpatient visits* | 3,165,890 (13.5) | 383,879 (12.9) | 2,782,011 (13.5) |
| Office visits | 13,478,810 (57.3) | 1,697,756 (57.1) | 11,781,054 (57.3) |
| Other† | 3,078,614 (13.1) | 407,639 (13.7) | 2,670,975 (13.0) |

ER, emergency room; IQR, interquartile range; N, number; SD, standard deviation

*These are outpatient visits still within the hospital system (on or off campus). †Other encompasses all places not captured in any of the other categories, e.g., school, pharmacy, birthing center, military treatment facility, home, etc.

# Table S5 STI testing patterns of all episodes over the different index years, stratified by age at index, among a) men and b) women

a)

|  | **All episodes***  **N** | **Episodes with CT/NG testing**†  **N (%**)‡ | | **Episodes with non-CT/NG testing only**  **N (%)** | | | | **Episodes with no testing**  **N (%)** | | | | |  |  |  |  |  |  |  |  |
| --- | --- | --- | --- | --- | --- | --- | --- | --- | --- | --- | --- | --- | --- | --- | --- | --- | --- | --- | --- | --- |
| **14–19 years-old at index** | | | | | | | | | | | | |  |  |  |  |  |  |  |  |
| **All episodes** | 233,699 | 73,738 (31.6) | | 116,444 (49.8) | | | | 43,517 (18.6) | | | | |  |  |  |  |  |  |  |  |
| **2010** | 19,065 | 3,977 (20.9) | | 10,666 (55.9) | | | | 4,422 (23.2) | | | | |  |  |  |  |  |  |  |  |
| **2011** | 20,392 | 4,655 (22.8) | | 11,310 (55.5) | | | | 4,427 (21.7) | | | | |  |  |  |  |  |  |  |  |
| **2012** | 27,640 | 7,248 (26.2) | | 14,896 (53.9) | | | | 5,496 (19.9) | | | | |  |  |  |  |  |  |  |  |
| **2013** | 25,706 | 7,065 (27.5) | | 13,820 (53.8) | | | | 4,821 (18.8) | | | | |  |  |  |  |  |  |  |  |
| **2014** | 28,251 | 8,700 (30.8) | | 14,265 (50.5) | | | | 5,286 (18.7) | | | | |  |  |  |  |  |  |  |  |
| **2015**§ | 25,768 | 8,485 (32.9) | | 12,378 (48.0) | | | | 4,905 (19.0) | | | | |  |  |  |  |  |  |  |  |
| **2016** | 26,490 | 9,527 (36.0) | | 12,347 (46.6) | | | | 4,616 (17.4) | | | | |  |  |  |  |  |  |  |  |
| **2017** | 24,715 | 9,490 (38.4) | | 11,174 (45.2) | | | | 4,051 (16.4) | | | | |  |  |  |  |  |  |  |  |
| **2018** | 23,338 | 9,600 (41.1) | | 10,182 (43.6) | | | | 3,556 (15.2) | | | | |  |  |  |  |  |  |  |  |
| **2019** | 12,334 | 4,991 (40.5) | | 5,406 (43.8) | | | | 1,937 (15.7) | | | | |  |  |  |  |  |  |  |  |
| **20–24 years-old at index** | | | | | | | | | | | | |  |  |  |  |  |  |  |  |
| **All episodes** | 271,356 | 120,274(44.3) | | 94,363 (34.8) | | | | 56,719 (20.9) | | | | |  |  |  |  |  |  |  |  |
| **2010** | 19,273 | 6,719 (34.9) | | 7,753 (40.2) | | | | 4,801 (24.9) | | | | |  |  |  |  |  |  |  |  |
| **2011** | 26,875 | 9,955 (37.0) | | 10,382 (38.6) | | | | 6,538 (24.3) | | | | |  |  |  |  |  |  |  |  |
| **2012** | 29,470 | 11,230 (38.1) | | 11,407 (38.7) | | | | 6,833 (23.2) | | | | |  |  |  |  |  |  |  |  |
| **2013** | 29,214 | 11,472 (39.3) | | 11,050 (37.8) | | | | 6,692 (22.9) | | | | |  |  |  |  |  |  |  |  |
| **2014** | 31,843 | 13,614 (42.8) | | 11,439 (35.9) | | | | 6,790 (21.3) | | | | |  |  |  |  |  |  |  |  |
| **2015**§ | 30,200 | 13,693 (45.3) | | 10,177 (33.7) | | | | 6,330 (21.0) | | | | |  |  |  |  |  |  |  |  |
| **2016** | 30,225 | 14,484 (47.9) | | 9,898 (32.7) | | | | 5,843 (19.3) | | | | |  |  |  |  |  |  |  |  |
| **2017** | 29,280 | 14,963(51.1) | | 9,004 (30.8) | | | | 5,313 (18.1) | | | | |  |  |  |  |  |  |  |  |
| **2018** | 29,486 | 15,855 (53.8) | | 8,662 (29.4) | | | | 4,969 (16.9) | | | | |  |  |  |  |  |  |  |  |
| **2019** | 15,490 | 8,289 (53.5) | | 4,591 (29.6) | | | | 2,610 (16.8) | | | | |  |  |  |  |  |  |  |  |
| **25–29 years-old at index** | | | | | | | | | | | | |  |  |  |  |  |  |  |  |
| **All episodes** | 216,369 | 82,737 (38.2) | | 87,097 (40.3) | | | | 46,535 (21.5) | | | | |  |  |  |  |  |  |  |  |
| **2010** | 19,900 | 5,758 (28.9) | | 9,115 (45.8) | | | | 5,027 (25.3) | | | | |  |  |  |  |  |  |  |  |
| **2011** | 21,958 | 6,870 (31.3) | | 9,781 (44.5) | | | | 5,307 (24.2) | | | | |  |  |  |  |  |  |  |  |
| **2012** | 22,752 | 7,339 (32.3) | | 10,154 (44.6) | | | | 5,259 (23.1) | | | | |  |  |  |  |  |  |  |  |
| **2013** | 21,272 | 7,253 (34.1) | | 9,146 (43.0) | | | | 4,873(22.9) | | | | |  |  |  |  |  |  |  |  |
| **2014** | 23,864 | 8,628 (36.2) | | 9,884 (41.4) | | | | 5,352(22.4) | | | | |  |  |  |  |  |  |  |  |
| **2015**§ | 23,209 | 9,052 (39.0) | | 9,060 (39.0) | | | | 5,097 (22.0) | | | | |  |  |  |  |  |  |  |  |
| **2016** | 23,716 | 9,904 (41.8) | | 9,138 (38.5) | | | | 4,674 (19.7) | | | | |  |  |  |  |  |  |  |  |
| **2017** | 23,240 | 10,420 (44.8) | | 8,330 (35.8) | | | | 4,490 (19.3) | | | | |  |  |  |  |  |  |  |  |
| **2018** | 23,735 | 11,224 (47.3) | | 8,230 (34.7) | | | | 4,281 (18.0) | | | | |  |  |  |  |  |  |  |  |
| **2019** | 12,723 | 6,289 (49.4) | | 4,259 (33.5) | | | | 2,175 (17.1) | | | | |  |  |  |  |  |  |  |  |
| **30–34 years-old at index** | | | | | | | | | | | | |  |  |  |  |  |  |  |  |
| **All episodes** | 227,150 | 66,541 (29.3) | | 110,211 (48.5) | | | | 50,398 (22.2) | | | | |  |  |  |  |  |  |  |  |
| **2010** | 24,251 | 5,105 (21.1) | | 12,917 (53.3) | | | | 6,229 (25.7) | | | | |  |  |  |  |  |  |  |  |
| **2011** | 25,805 | 5,903 (22.9) | | 13,643 (52.9) | | | | 6,259 (24.3) | | | | |  |  |  |  |  |  |  |  |
| **2012** | 25,547 | 6,126 (24.0) | | 13,351 (52.3) | | | | 6,070 (23.8) | | | | |  |  |  |  |  |  |  |  |
| **2013** | 23,229 | 6,039 (26.0) | | 11,881 (51.1) | | | | 5,309 (22.9) | | | | |  |  |  |  |  |  |  |  |
| **2014** | 24,657 | 6,891 (27.9) | | 12,246 (49.7) | | | | 5,520 (22.4) | | | | |  |  |  |  |  |  |  |  |
| **2015**§ | 22,845 | 6843 (30.0) | | 10,871(47.6) | | | | 5,131 (22.5) | | | | |  |  |  |  |  |  |  |  |
| **2016** | 23,345 | 7,747 (33.2) | | 10,794 (46.2) | | | | 4,804 (20.6) | | | | |  |  |  |  |  |  |  |  |
| **2017** | 21,980 | 7,791 (35.4) | | 9,820 (44.7) | | | | 4,369 (19.9) | | | | |  |  |  |  |  |  |  |  |
| **2018** | 23,025 | 8,932 (38.8%) | | 9,698 (42.1) | | | | 4,395(19.1) | | | | |  |  |  |  |  |  |  |  |
| **2019** | 12,466 | 5,164 (41.4%) | | 4,990 (40.0) | | | | 2,312 (18.5) | | | | |  |  |  |  |  |  |  |  |
| **35–39 years-old at index** | | | | | | | | | | | | |  |  |  |  |  |  |  |  |
| **All episodes** | 243,434 | 54,273 (22.3) | | 133,457 (54.8) | | | | 55,704 (22.9) | | | | |  |  |  |  |  |  |  |  |
| **2010** | 28,842 | 4,504 (15.6) | | 16,926 (58.7) | | | | 7,412 (25.7) | | | | |  |  |  |  |  |  |  |  |
| **2011** | 28,664 | 4,807 (16.8) | | 16,833(58.7) | | | | 7,024 (24.5) | | | | |  |  |  |  |  |  |  |  |
| **2012** | 27,612 | 5,033 (18.2) | | 15,989 (57.9) | | | | 6,590(23.9) | | | | |  |  |  |  |  |  |  |  |
| **2013** | 25,283 | 4,901 (19.4) | | 14,557 (57.6) | | | | 5,825 (23.0) | | | | |  |  |  |  |  |  |  |  |
| **2014** | 26,107 | 5,546 (21.2) | | 14,556 (55.8) | | | | 6,005 (23.0) | | | | |  |  |  |  |  |  |  |  |
| **2015**§ | 24,261 | 5,704 (23.5) | | 12,960 (53.4) | | | | 5,597 (23.1) | | | | |  |  |  |  |  |  |  |  |
| **2016** | 24,156 | 6,186 (25.6) | | 12,812 (53.0) | | | | 5,158 (21.4) | | | | |  |  |  |  |  |  |  |  |
| **2017** | 22,850 | 6,464 (28.3) | | 11,616 (50.8) | | | | 4,770 (20.9) | | | | |  |  |  |  |  |  |  |  |
| **2018** | 23,394 | 7,129 (30.5) | | 11,470 (49.0) | | | | 4,795 (20.5) | | | | |  |  |  |  |  |  |  |  |
| **2019** | 12,265 | 3,999 (32.6) | | 5,738 (46.8) | | | | 2,528 (20.6) | | | | |  |  |  |  |  |  |  |  |
| **40**–64 **years-old at index** | | | | | | | | | | | | |  |  |  |  |  |  |  |  |
| **All episodes** | 1,781,675 | 139,614 (7.8) | | 1,250,444 (70.2) | | | | 391,617 (22.0) | | | | |  |  |  |  |  |  |  |  |
| **2010** | 213,995 | 11,361 (5.3) | | 152,871 (71.4) | | | | 49,763(23.3) | | | | |  |  |  |  |  |  |  |  |
| **2011** | 219,187 | 12,643 (5.8) | | 157,427 (71.8) | | | | 49,117 (22.4) | | | | |  |  |  |  |  |  |  |  |
| **2012** | 208,574 | 13,538 (6.5) | | 149,197 (71.5) | | | | 45,839 (22.0) | | | | |  |  |  |  |  |  |  |  |
| **2013** | 196,656 | 13,612 (6.9) | | 140,971 (71.7) | | | | 42,073 (21.4) | | | | |  |  |  |  |  |  |  |  |
| **2014** | 195,941 | 14,487 (7.4) | | 139,184 (71.0) | | | | 42,270 (21.6) | | | | |  |  |  |  |  |  |  |  |
| **2015**§ | 175,412 | 14,456 (8.2) | | 121,967 (69.5) | | | | 38,989 (22.2) | | | | |  |  |  |  |  |  |  |  |
| **2016** | 170,723 | 15,526 (9.1) | | 117,979 (69.1) | | | | 37,218 (21.8) | | | | |  |  |  |  |  |  |  |  |
| **2017** | 160,669 | 16,178 (10.1) | | 109,727 (68.3) | | | | 34,764 (21.6) | | | | |  |  |  |  |  |  |  |  |
| **2018** | 158,506 | 17,808 (11.2) | | 106,786 (67.4) | | | | 33,912 (21.4) | | | | |  |  |  |  |  |  |  |  |
| **2019** | 82,012 | 10,005 (12.2) | | 54,335 (66.3) | | | | 17,672 (21.5) | | | | |  |  |  |  |  |  |  |  |
|  |  |  |  |  | |  | | |  | |  | |  | |  | |  | |  |  |
| b) | |  |  |  |  | |  | | |  | |  | |  | |  | |  | |  |
|  | **All episodes***  **N** | **Episodes with CT/NG testing**†  **N (%)**‡ | | **Episodes with non-CT/NG testing only**  **N (%)** | | | | **Episodes with no testing**  **N (%)** | | | | |  |  |  |  |  |  |  |  |
| **14–19 years-old at index** | | | | | | | | | | | | |  |  |  |  |  |  |  |  |
| **All episodes** | 1,942,852 | 490,762 (25.3) | | 1,215,844 (62.6) | | | | 236,246 (12.2) | | | | |  |  |  |  |  |  |  |  |
| **2010** | 154,310 | 30,471 (19.7) | | 101,264 (65.6) | | | | 22575 (14.6) | | | | |  |  |  |  |  |  |  |  |
| **2011** | 164,716 | 33,901 (20.6) | | 108,101 (65.6) | | | | 22,714 (13.8) | | | | |  |  |  |  |  |  |  |  |
| **2012** | 243,552 | 59,900(24.6) | | 152,123 (62.5) | | | | 31,529 (12.9) | | | | |  |  |  |  |  |  |  |  |
| **2013** | 227,459 | 55,938 (24.6) | | 143,323 (63.0) | | | | 28,198 (12.4) | | | | |  |  |  |  |  |  |  |  |
| **2014** | 244,785 | 62,101 (25.4) | | 152,791 (62.4) | | | | 29,893(12.2) | | | | |  |  |  |  |  |  |  |  |
| **2015**§ | 222,918 | 57,587 (25.8) | | 138,450 (62.1) | | | | 26,881 (12.1) | | | | |  |  |  |  |  |  |  |  |
| **2016** | 217,282 | 57,813 (26.6) | | 135,273 (62.3) | | | | 24,196 (11.1) | | | | |  |  |  |  |  |  |  |  |
| **2017** | 196,456 | 54,349 (27.7) | | 120,376 (61.3) | | | | 21,731 (11.1) | | | | |  |  |  |  |  |  |  |  |
| **2018** | 182,994 | 53,010 (29.0) | | 110,711 (60.5) | | | | 19,273 (10.5) | | | | |  |  |  |  |  |  |  |  |
| **2019** | 88,380 | 25,692 (29.1) | | 53,432 (60.5) | | | | 9,256 (10.5) | | | | |  |  |  |  |  |  |  |  |
| **20–24 years-old at index** | | | | | | | | | | | | |  |  |  |  |  |  |  |  |
| **All episodes** | 2,779,432 | 870,400 (31.3) | | 1,531,524 (55.1) | | | | 377,508 (13.6) | | | | |  |  |  |  |  |  |  |  |
| **2010** | 189,192 | 48,391 (25.6) | | 110,363 (58.3) | | | | 30,438 (16.1) | | | | |  |  |  |  |  |  |  |  |
| **2011** | 244,003 | 65,397 (26.8) | | 140,684 (57.7) | | | | 37,922 (15.5) | | | | |  |  |  |  |  |  |  |  |
| **2012** | 328,617 | 96,703 (29.4) | | 183,652 (55.9) | | | | 48,262 (14.7) | | | | |  |  |  |  |  |  |  |  |
| **2013** | 320,797 | 97,189 (30.3) | | 178,954 (55.8) | | | | 44,654 (13.9) | | | | |  |  |  |  |  |  |  |  |
| **2014** | 359,829 | 114,660 (31.9) | | 196,880 (54.7) | | | | 48,289 (13.4) | | | | |  |  |  |  |  |  |  |  |
| **2015**§ | 332,168 | 107,091 (32.2) | | 180,816 (54.4) | | | | 44,261 (13.3) | | | | |  |  |  |  |  |  |  |  |
| **2016** | 309,176 | 102,472 (33.1) | | 168,446 (54.5) | | | | 38,258 (12.4) | | | | |  |  |  |  |  |  |  |  |
| **2017** | 284,488 | 95,232 (33.5) | | 153,817 (54.1) | | | | 35,439 (12.5) | | | | |  |  |  |  |  |  |  |  |
| **2018** | 273,797 | 94,036 (34.3) | | 146,109 (53.4) | | | | 33,652 (12.3) | | | | |  |  |  |  |  |  |  |  |
| **2019** | 137,365 | 49,229 (35.8) | | 71,803 (52.3) | | | | 16,333 (11.9) | | | | |  |  |  |  |  |  |  |  |
| **25–29 years-old at index** | | | | | | | | | | | | |  |  |  |  |  |  |  |  |
| **All episodes** | 2,385,694 | 671,539 (28.1) | | 1,372,264 (57.5) | | | | 341,891 (14.3) | | | | |  |  |  |  |  |  |  |  |
| **2010** | 203,477 | 43,578 (21.4) | | 127,888(62.9) | | | | 32,011 (15.7) | | | | |  |  |  |  |  |  |  |  |
| **2011** | 213,966 | 47,650 (22.3) | | 134,009 (62.6) | | | | 32,307 (15.1) | | | | |  |  |  |  |  |  |  |  |
| **2012** | 262,110 | 67,285 (25.7) | | 156,662 (59.8) | | | | 38,163 (14.6) | | | | |  |  |  |  |  |  |  |  |
| **2013** | 251,858 | 67,456 (26.8) | | 148,594 (59.0) | | | | 35,808 (14.2) | | | | |  |  |  |  |  |  |  |  |
| **2014** | 296,444 | 85,982 (29.0) | | 168,409 (56.8) | | | | 42,053 (14.2) | | | | |  |  |  |  |  |  |  |  |
| **2015**§ | 277,799 | 82,614 (29.7) | | 155,590 (56.0) | | | | 39,595(14.3) | | | | |  |  |  |  |  |  |  |  |
| **2016** | 268,319 | 81,985 (30.6) | | 149,796 (55.8) | | | | 36,538(13.6) | | | | |  |  |  |  |  |  |  |  |
| **2017** | 248,638 | 76,656 (30.8) | | 137,309 (55.2) | | | | 34,673 (13.9) | | | | |  |  |  |  |  |  |  |  |
| **2018** | 239,172 | 76,645 (32.0) | | 129,034 (54.0) | | | | 33,493 (14.0) | | | | |  |  |  |  |  |  |  |  |
| **2019** | 123,911 | 41,688 (33.6) | | 64,973 (52.4) | | | | 17,250 (13.9) | | | | |  |  |  |  |  |  |  |  |
| **30–34 years-old at index** | | | | | | | | | | | | |  |  |  |  |  |  |  |  |
| **All episodes** | 2,279,176 | 519,450 (22.8) | | 1,420,423 (62.3) | | | | 339,303 (14.9) | | | | |  |  |  |  |  |  |  |  |
| **2010** | 229,295 | 40,324 (17.6) | | 152,700 (66.6) | | | | 36,271 (15.8) | | | | |  |  |  |  |  |  |  |  |
| **2011** | 237,728 | 43,593 (18.3) | | 158,538 (66.7) | | | | 35,597 (15.0) | | | | |  |  |  |  |  |  |  |  |
| **2012** | 261,847 | 54,475 (20.8) | | 168,584 (64.4) | | | | 38,788 (14.8) | | | | |  |  |  |  |  |  |  |  |
| **2013** | 248,200 | 53,631 (21.6) | | 158,527 (63.9) | | | | 36,042 (14.5) | | | | |  |  |  |  |  |  |  |  |
| **2014** | 274,333 | 64,310 (23.4) | | 169,471 (61.8) | | | | 40,552 (14.8) | | | | |  |  |  |  |  |  |  |  |
| **2015**§ | 274,370 | 60,029 (24.3) | | 150,470 (60.8) | | | | 36,871 (14.9) | | | | |  |  |  |  |  |  |  |  |
| **2016** | 235,274 | 58,903 (25.0) | | 142,631 (60.6) | | | | 33,740 (14.3) | | | | |  |  |  |  |  |  |  |  |
| **2017** | 218,226 | 55,161(25.3) | | 130,713 (59.9) | | | | 32,352 (14.8) | | | | |  |  |  |  |  |  |  |  |
| **2018** | 214,721 | 57,417 (26.7) | | 125,306 (58.4) | | | | 31,998 (14.9) | | | | |  |  |  |  |  |  |  |  |
| **2019** | 112,182 | 31,607 (28.2) | | 63,483 (56.6) | | | | 17,092 (15.2) | | | | |  |  |  |  |  |  |  |  |
| **35–39 years-old at index** | | | | | | | | | | | | |  |  |  |  |  |  |  |  |
| **All episodes** | 2,088,773 | 382,113 (18.3) | | 1,379,599 (66.0) | | | | 327,061 (15.7) | | | | |  |  |  |  |  |  |  |  |
| **2010** | 225,393 | 32,059 (14.2) | | 156,621 (69.5) | | | | 36,713 (16.3) | | | | |  |  |  |  |  |  |  |  |
| **2011** | 228,466 | 33,610 (14.7) | | 159,214 (69.7) | | | | 35,642 (15.6) | | | | |  |  |  |  |  |  |  |  |
| **2012** | 237,035 | 38,985 (16.4) | | 161,425 (68.1) | | | | 36,625 (15.5) | | | | |  |  |  |  |  |  |  |  |
| **2013** | 224,441 | 39,119 (17.4) | | 151,383 (67.4) | | | | 33,939 (15.1) | | | | |  |  |  |  |  |  |  |  |
| **2014** | 244,445 | 46,291 (18.9) | | 159,900 (65.4) | | | | 38,254 (15.6) | | | | |  |  |  |  |  |  |  |  |
| **2015**§ | 223,833 | 43,826 (19.6) | | 144,301 (64.5) | | | | 35,706 (16.0) | | | | |  |  |  |  |  |  |  |  |
| **2016** | 213,508 | 43,048 (20.2) | | 138,125 (64.7) | | | | 32,335 (15.1) | | | | |  |  |  |  |  |  |  |  |
| **2017** | 198,942 | 40,713 (20.5) | | 127,058 (63.9) | | | | 31,171 (15.7) | | | | |  |  |  |  |  |  |  |  |
| **2018** | 194,151 | 41,794 (21.5) | | 121,530 (62.6) | | | | 30,827 (15.9) | | | | |  |  |  |  |  |  |  |  |
| **2019** | 98,559 | 22,668 (23.0) | | 60,042(60.9) | | | | 15,849 (16.1) | | | | |  |  |  |  |  |  |  |  |
| **40**–64 **years-old at index** | | | | | | | | | | | | |  |  |  |  |  |  |  |  |
| **All episodes** | 9,088,202 | 674,178 (7.4) | | 6,977,734 (76.8) | | | | 1,436,290 (15.8) | | | | |  |  |  |  |  |  |  |  |
| **2010** | 1,056,342 | 61,037 (5.8) | | 822,047 (77.8) | | | | 173,258 (16.4) | | | | |  |  |  |  |  |  |  |  |
| **2011** | 1,101,320 | 66,628 (6.0) | | 861,032 (78.2) | | | | 173,660 (15.8) | | | | |  |  |  |  |  |  |  |  |
| **2012** | 1,064,931 | 71,432 (6.7) | | 826,759 (77.6) | | | | 166,740 (15.7) | | | | |  |  |  |  |  |  |  |  |
| **2013** | 1,015,226 | 73,425 (7.2) | | 786,510 (77.5) | | | | 155,291 (15.3) | | | | |  |  |  |  |  |  |  |  |
| **2014** | 1,041,386 | 79,332 (7.6) | | 795,020 (76.3) | | | | 167,034 (16.0) | | | | |  |  |  |  |  |  |  |  |
| **2015**§ | 927,952 | 73,526 (7.9) | | 704,482 (75.9) | | | | 149,944 (16.2) | | | | |  |  |  |  |  |  |  |  |
| **2016** | 871,459 | 70,453 (8.1) | | 668,203 (76.7) | | | | 132,803 (15.2) | | | | |  |  |  |  |  |  |  |  |
| **2017** | 806,720 | 66,700 (8.3) | | 614,543 (76.2) | | | | 125,477 (15.6) | | | | |  |  |  |  |  |  |  |  |
| **2018** | 799,106 | 71,328 (8.9) | | 601,406 (75.3) | | | | 126,372 (15.8) | | | | |  |  |  |  |  |  |  |  |
| **2019** | 403,760 | 40,317 (10.0) | | 297,732 (73.7) | | | | 65,711 (16.3) | | | | |  |  |  |  |  |  |  |  |
|  | | | | | | | | | | | | | | | | | | | | |

*Testing within 1–3 days of index date and/or any return visits within each episode (date of visit as day 1). †Includes episodes that received CT/NG testing only and episodes that received CT/NG testing as well as other non-CT/NG testing. ‡All percentages are row percentages. §IBM MarketScan Research Databases started to lose several contributors of data after 2015.

CT, *Chlamydia trachomatis*; NG, *Neisseria gonorrhoeae*
